# Supplementary material for: Bacterial Communities in the Rhizosphere at Different Growth Stages of Maize Cultivated in Soil Under Conventional and Conservation Agricultural Practices
Source: Microbiol Spectr. 2022 Mar 7;10(2):e01834-21. doi: 10.1128/spectrum.01834-21 (PMC9049951; doi:10.1128/spectrum.01834-21)
Supplement: SUPPLEMENTAL FILE 1 — Fig. S1 to S4. Download SPECTRUM01834-21_Supp_1_seq12.pdf, PDF file, 1.3 MB [file spectrum01834-21_supp_1_seq12.pdf]

Fig.S1

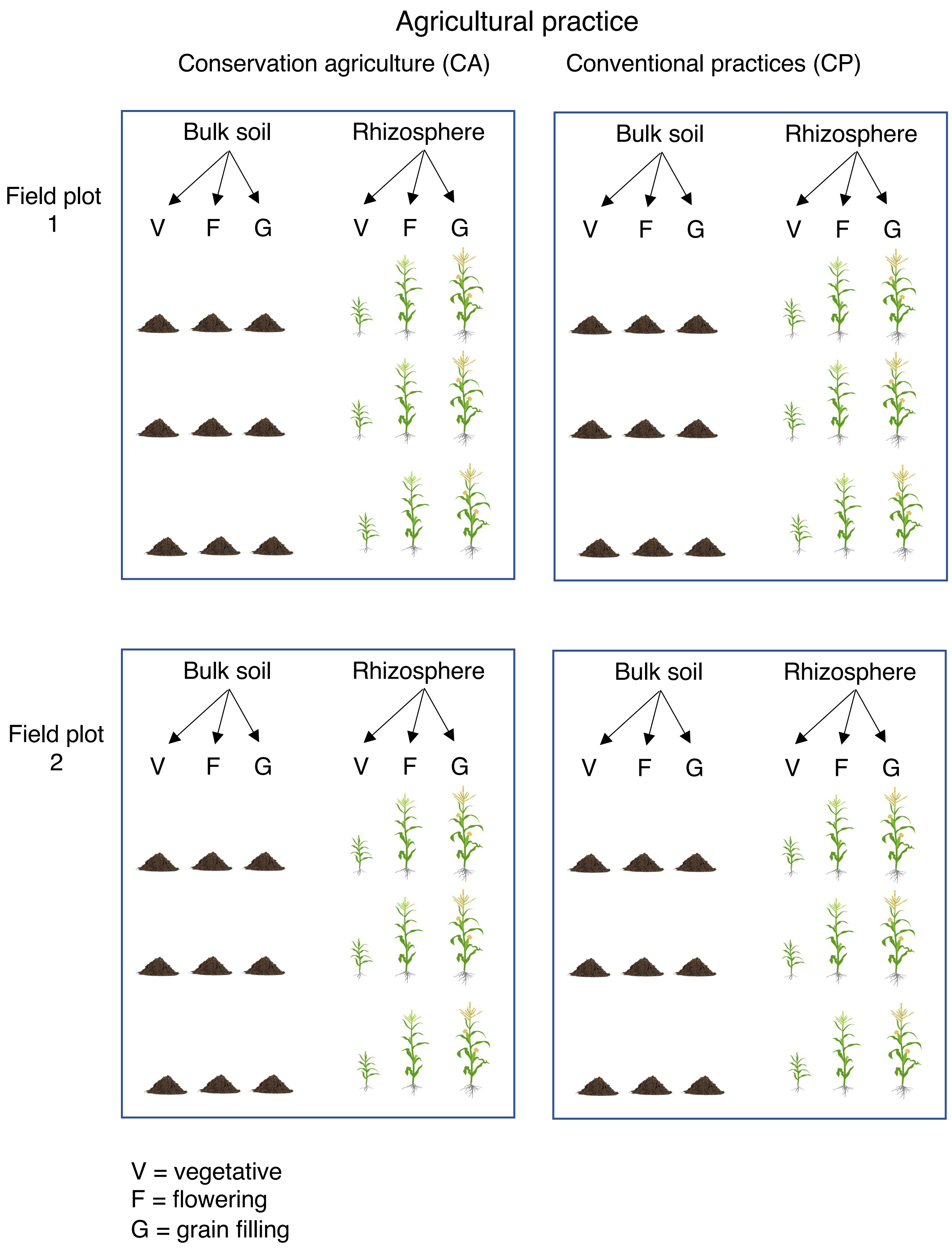

Figure S1. Experimental design

Fig.S2

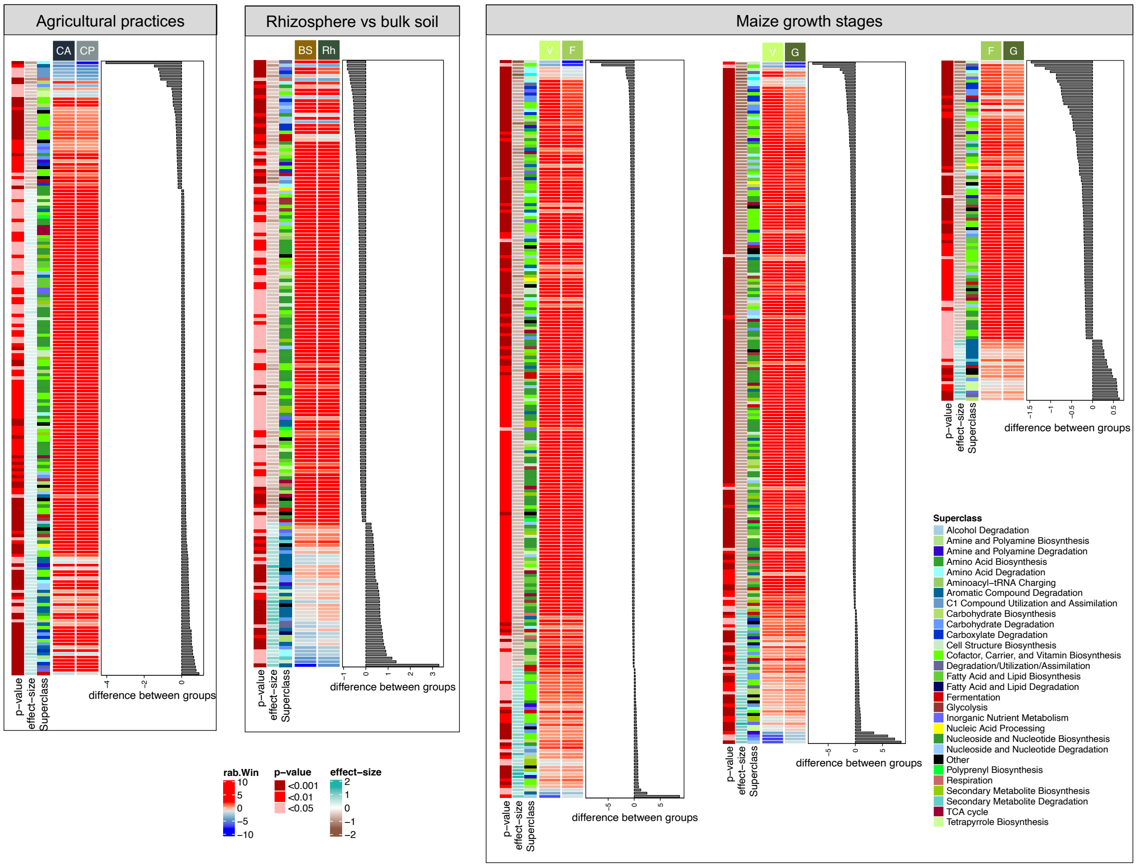

Figure S2. Differentially abundant predicted functions of soil bacterial communities as determined by an ANOVA-like differential expression tool for compositional data and Benjamini-Hochberg sequential correction. Effect of agricultural practices, i.e. conservation agriculture (CA) and conventional practices (CP), of maize (*Zea mays* L.) rhizosphere (Rh) versus the bulk soil (BS) and the maize rhizosphere along the vegetative (V), flowering (F) and grain filling (G) growth stages on soil bacterial communities. Functional predictions were determined with the ancestral reconstruction algorithm in PICRUSt. Median centered log-ratio (*clr*) transformed frequencies, *p*-values and the effect were plotted as heat-map and the median difference between species as bar-plot.

Fig.S3

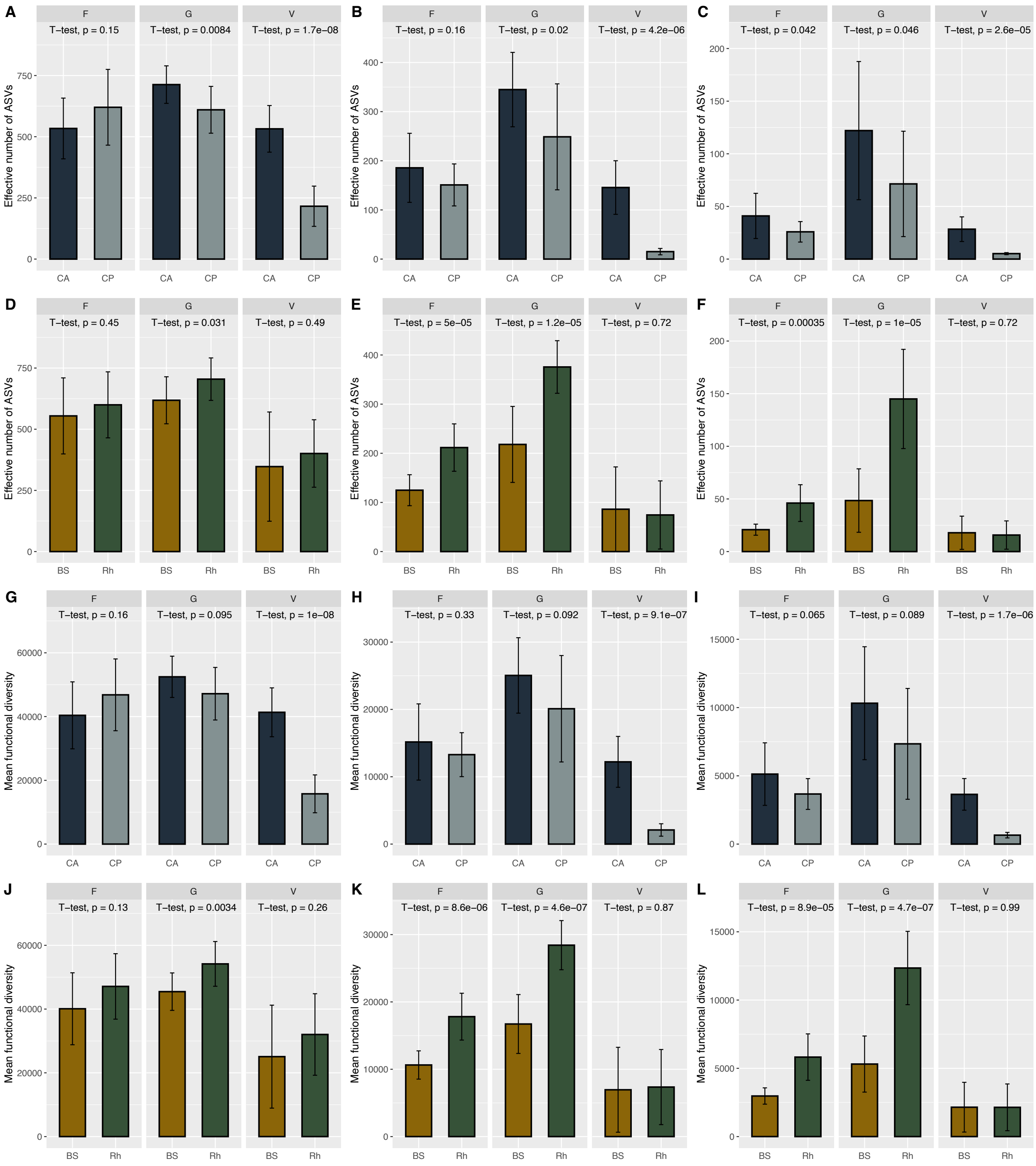

Figure S3. Hill numbers of alpha taxonomic diversity and mean functional diversity. The bar height indicates the mean for each growth stage and the error line shows the standard error of the mean. Significant differences between treatments were determined by a t-test.

Fig. S4

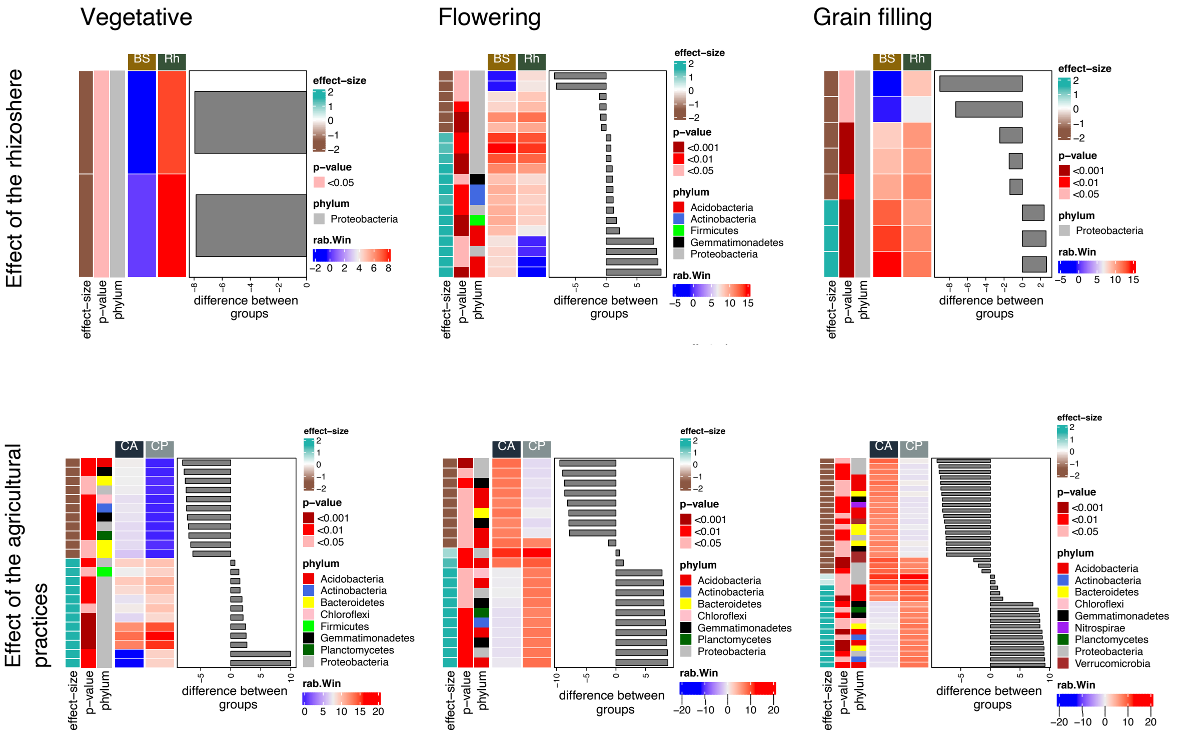

Figure S4. Differentially abundant amplicon sequence variants (ASVs) as determined by an ANOVA-like differential expression tool for compositional data and Benjamini-Hochberg sequential correction. Effect of agricultural practices, i.e. conservation agriculture (CA) and conventional practices (CP), of maize (*Zea mays* L.) rhizosphere (Rh) versus the bulk soil (BS) during the vegetative, flowering and grain filling growth stages on soil bacterial communities. Median centered log-ratio (*clr*) transformed frequencies, *p*-values and the effect were plotted as heat-map and the median difference between species as bar-plot.
